# The effects of intravitreal sodium iodate injection on retinal degeneration following vitrectomy in rabbits

**DOI:** 10.1038/s41598-019-52172-y

**Published:** 2019-10-30

**Authors:** So Min Ahn, Jungryul Ahn, Seongkwang Cha, Cheolmin Yun, Tae Kwann Park, Young-Jin Kim, Yong Sook Goo, Seong-Woo Kim

**Affiliations:** 1 Department of Ophthalmology, Korea University College of Medicine, Seoul, Korea; 2 Department of Physiology, Chungbuk National University School of Medicine, Cheongju, Korea; 3 Department of Ophthalmology, Bucheon Hospital, Soonchunhyang University College of Medicine, Bucheon, Korea; 4 Medical Device Development Center, Osong Medical Innovation Foundation, Cheongju, Korea

**Keywords:** Experimental models of disease, Retinal diseases

## Abstract

We sought to develop and characterize outer retinal degeneration induced by intravitreal injection of sodium iodate (SI) after vitrectomy in rabbits. To determine the effective dose of SI, the right eyes of 19 male New Zealand white rabbits received an intravitreal injection of SI or sham. Based on the dose-dependence results, 0.4 mg of SI in 0.05 mL of total volume was injected into the right eyes of 10 rabbits at two weeks after vitrectomy. In the dose-dependence study, localized retinal atrophy was observed with 0.3- and 0.4-mg SI injections without vitrectomy. Severe and diffuse retinal atrophy was identified by spectral-domain optical coherence tomography (SD-OCT) at one month after a 0.5-mg SI injection following vitrectomy. In the second experiment, 0.4 mg of SI in 0.05 mL was injected, and the severity of outer retinal degeneration was graded as one of two types according to electroretinography (ERG) response change. There was no response on ERG in complete retinal degeneration, 30% of all 10 rabbits. Intravitreal injection of 0.4 mg of SI into vitrectomized rabbit eyes induces diffuse outer retinal degeneration, and the degree of retinal degeneration can be evaluated through *in vivo* ophthalmic examination.

## Introduction

Retinal degeneration, which includes conditions such as retinitis pigmentosa (RP), choroideremia, and geographic atrophy (GA) of age-related macular degeneration (ARMD), is the main cause of irreversible vision loss and greatly affects quality of life. RP is the most common inherited retinal dystrophy and leads to irreversible vision loss. Initial degeneration due to RP occurs in the photoreceptors, and inner retinal thickness is gradually decreased in advanced-stage RP^1–3^. Visual prosthetics such as retinal implants have been developed for treatment of retinal degeneration due to advances in electronic device technology and biomaterials^4,5^. Recently, implantation of visual prosthetics has been performed in humans. Therefore, to further develop and refine such medical devices, larger experimental animal models (e.g., dogs, pigs, cats, rabbits) with specific loss of photoreceptors are inevitably needed.

The retinotoxin sodium iodate (SI) is an oxidizing compound toxic to retinal pigment epithelial (RPE) cells, with secondary effects on photoreceptors and the choriocapillaris^6^. Specifically, SI primarily induces necrosis in RPE cells^7,8^, which is followed by choriocapillaris atrophy^9^ and panretinal degeneration^8,10^. In addition to these effects on RPE cells and photoreceptors, SI also provokes necrosis of the inner retina^8,11^. SI induces the production of reactive oxygen species, which contribute to damage in the RPE cells^12^. SI retinal toxicity has been demonstrated in many different mammalian species, including sheep^7^, rabbits^13,14^, rats^10,15^, and mice^6,11,16^, with varying doses and routes of administration. Most studies have used relatively high doses of SI (50–100 mg/kg) and have reported rapid RPE damage characterized by defragmentation and loss of RPE cell nuclei. Systemic application of SI leads not only to bilateral retinal degeneration, but also to reduced general health of the experimental animals. Systemic intoxication of SI after systemic administration includes gastrointestinal problems such as diarrhea, general weakness, and convulsion^17,18^. A high dose of SI was found to be lethal in experimental animals^17,18^. Therefore, local administration of SI is required to avoid its systemic effects.

In the present research, we attempted to induce unilateral diffuse homogeneous outer retinal degeneration of the whole retina by intravitreal administration of SI in rabbits. We hypothesized that this approach would avoid the known systemic side effects. The primary objective of this study was to elucidate the necessary effects of vitrectomy and the proper intravitreal SI dose following vitrectomy to induce diffuse homogeneous outer retinal degeneration in rabbits. Secondarily, we evaluated the ability of the determined dose of intravitreally injected SI to induce diffuse outer retinal degeneration.

## Results

### Retinal imaging in the dose-dependence study of sodium iodate without pars plana vitrectomy

**Figure 1:**
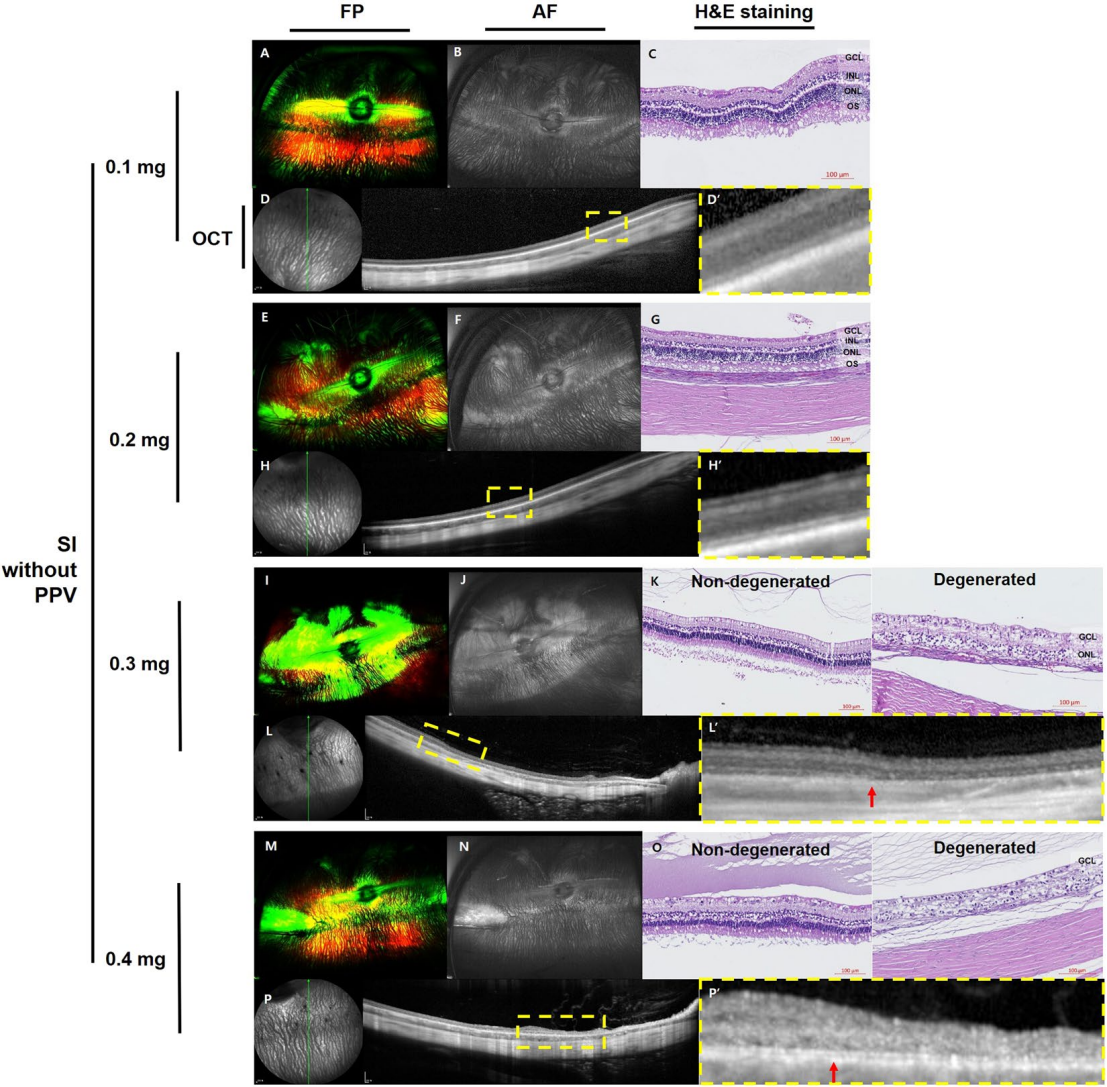
Ultra-wide-field color FP and AF images, histology with H&E staining, and SD-OCT images at one month after intravitreal injection of SI without vitrectomy. One month after injection, no significant changes were observed in FP, AF, histology, or OCT images of rabbit eyes injected with 0.1 mg of SI (**A–D**). Focal hyperautofluorescent areas were observed in eyes injected with 0.2 mg (**F**), 0.3 mg (**J**), and 0.4 mg (**N**) of SI without vitrectomy. Retinal atrophy was seen via H&E staining and SD-OCT following injection with 0.3 mg (**K,L**) and 0.4 mg (**O,P**) of SI. Histology with H&E staining in 0.3 mg and 0.4 mg of SI injection showed not only the photoreceptor layer but also all layers of the retina appeared normal in the non-degenerated area, but loss of the photoreceptor layer and disruption of retinal layers were found in the degenerated area (**K,O**). In the magnified OCT image (L’,P’, dashed-line box in **L,P**), the borderline between the degenerated area and non-degenerated area was clearly delineated (L’,P’, red arrow) and the degeneration of the outer retina and retinal thinning were detected in the degenerated area (L’,P’, right side of the red arrow). However, in non-degenerated area, whole retinal layers were relatively intact in OCT image (L’,P’, left side of the red arrow). The green line on infrared FP shows the plane where the SD-OCT images were collected (**D,H,L,P**). Magnified SD-OCT images are shown (D’,H’,L’,P’; dashed-line box in **D,H,L,P**).

At one month after SI injection, no significant changes were observed in fundus photography (FP), fundus autofluorescence (AF), histology with hematoxylin and eosin (H&E) staining, or spectral-domain optical coherence tomography (SD-OCT) images of rabbit eyes injected with 0.1 mg of SI (Fig. 1A–D). Localized hyper-autofluorescent areas were observed in eyes injected with 0.2 mg, 0.3 mg, or 0.4 mg of SI without vitrectomy (Fig. 1F,J,N, respectively). Both non-degenerated retina and degenerated retina were observed by histology and SD-OCT in the rabbit eyes injected with 0.3 mg (Fig. 1K,L) or 0.4 mg (Fig. 1O,P) of SI. Disruption of the outer retina and decrease in retinal thickness were observed in degenerated retina.

### Retinal imaging in the dose-dependence study of sodium iodate after pars plana vitrectomy

**Figure 2:**
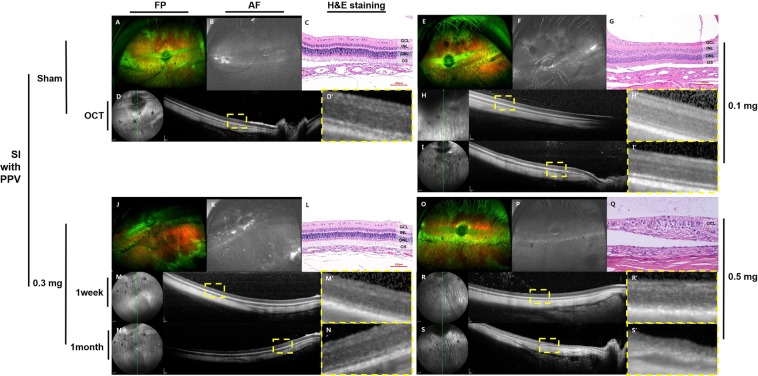
Ultra-wide-field color FP and AF images, histology with H&E staining, and OCT images after intravitreal injection of SI after vitrectomy. One month after SI injection, no significant changes in autofluorescence were observed by ultra-wide-field AF (**F,K,P**). No significant changes were observed by OCT and H&E staining following the sham (**C,D**), and 0.1-mg SI (**G,H,I**) injections after vitrectomy. In eyes injected with 0.3 mg of SI after vitrectomy, which showed selective outer retinal degeneration at one week and one month on SD-OCT (**M,N**), the outer retinal layer was distinguished from the inner nuclear layer by H&E staining in the area where the retinal layer remained relatively intact on SD-OCT (**L**). In eyes injected with 0.5 mg of SI after vitrectomy, severe retinal atrophy was observed on H&E staining (**Q**) and degenerative changes of the retina which showed at one week on OCT (R) became worse at one month on OCT (**S**). The green line on infrared FP shows the plane where the SD-OCT images were collected (**D,H,I,M,N,R,S**). Magnified OCT images are shown (D’,H’,I’,M’,N’,R’,S’; dashed-line box in **D,H,I,M,N,R,S**).

At one month after SI injection, no localized hyper- or hypo autofluorescence was observed by ultra-wide-field color FP or AF (Fig. 2).

Additionally, no significant retinal changes were observed with SD-OCT in eyes injected with sham at one month, and 0.1 mg of SI at one week and one month (Fig. 2D,H,I). However, in the retina of rabbit eyes injected with 0.5 mg of SI, degenerative changes of the outer retina were observed at one week (Fig. 2R) and degenerative changes became worse for one month (Fig. 2S). The interlayer boundary of the inner and outer retina was unclear, and layers of the outer retina were replaced with multiple hyper-reflective materials at one month (Fig. 2S). In one of the three rabbit eyes injected with 0.3 mg of SI, degenerative changes of the outer retina were observed at both one week and one month, while the inner retina remained normal (Fig. 2M,N).

An example of histological examination by H&E staining at one month is presented in Fig. 2. In the eye injected with 0.5 mg of SI after vitrectomy, which resulted in severe degenerative changes of the retina observed by SD-OCT, the layers of the retina were disrupted and not distinguishable (Fig. 2Q). The nuclei of the inner nuclear layer (INL) and outer nuclear layer (ONL) were mixed and scattered, and there was a loss of the photoreceptor layer. In the eyes injected with 0.3 mg of SI after vitrectomy with selective outer retinal degeneration on SD-OCT, the outer retinal layer was distinguishable from the INL in the area that remained relatively intact on SD-OCT (Fig. 2L). Following sham and 0.1-mg SI injections after vitrectomy, no significant changes were observed in H&E staining (Fig. 2C,G).

### Retinal degeneration induced by 0.4-mg sodium iodate injection with vitrectomy

**Figure 3:**
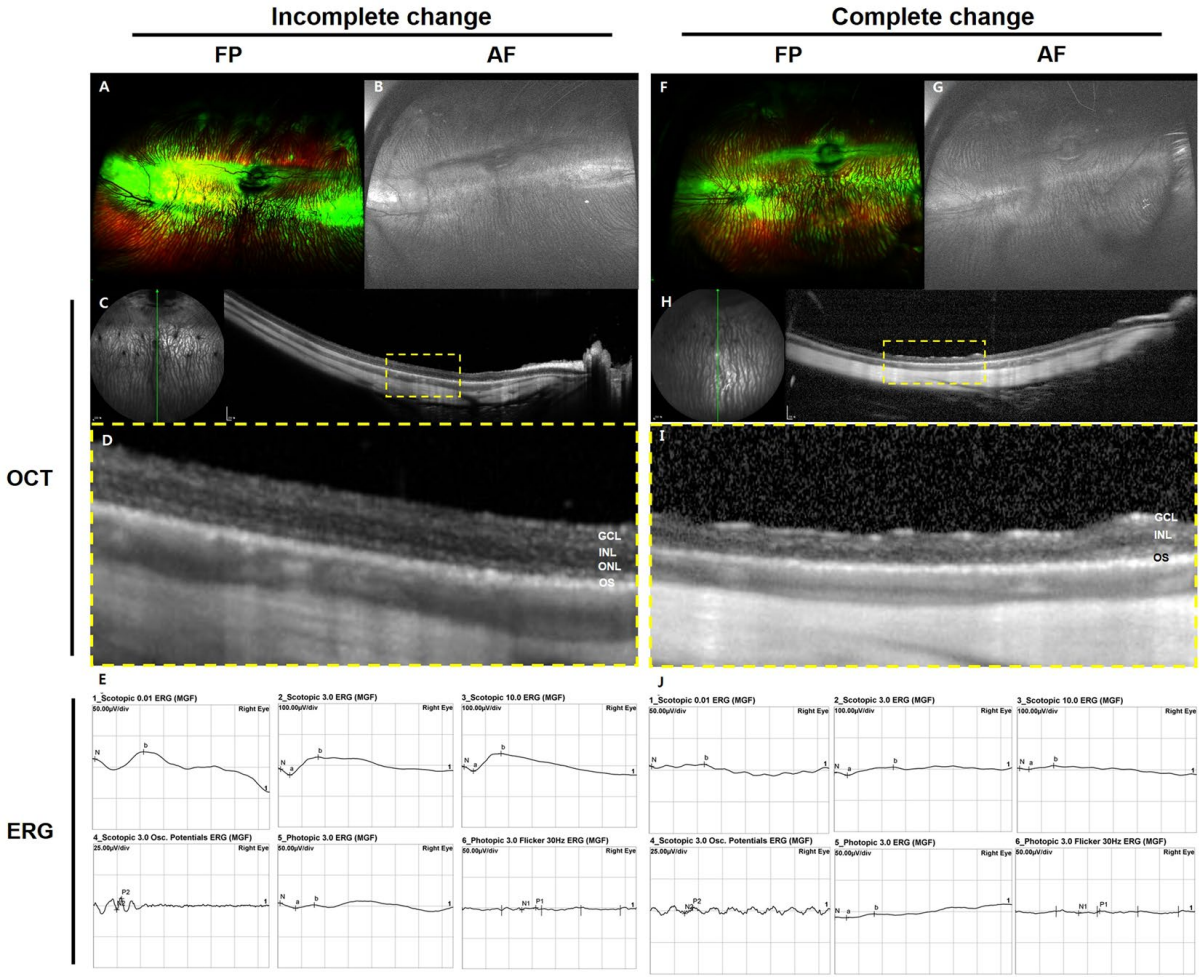
Ultra-wide-field color FP and AF images, SD-OCT, and ERG at six weeks after intravitreal injection of 0.4 mg of SI with vitrectomy. In eyes with incomplete and complete retinal degeneration, FP and AF images did not show any localized degenerative change (**A,B,F,G**). In eyes with incomplete retinal degeneration, the IS/OS line and external limiting membraine (ELM) could not be differentiated, and a thickened single line was observed on SD-OCT (**C,D**). There was partially remnant response according to ERG (**E**). In eyes with complete retinal degeneration, those with diffuse degeneration on OCT (**H,I**) had no response according to ERG (**J**). The green line on infrared FP shows the plane wherein the SD-OCT images were collected (**C,H**). Magnified OCT images are shown (**D,I**; dashed-line box in **C,H**).

Based on the dose-dependence study, 0.4 mg of SI in 0.05 mL of total volume was selected for further evaluation. Six weeks after the 0.4-mg SI injection, the severity of outer retinal degeneration was graded into two types according to change in OCT images and electroretinography (ERG) response (Fig. 3). For animals classified as having incomplete or complete retinal degeneration, the FP images showed degenerative changes (Fig. 3A,F), the AF images showed hyper-autofluorescence (Fig. 3B,G), and the ERG response was abnormal (Fig. 3E,J). FP and AF images did not show any localized degenerative change. Seven of the 10 rabbits showed incomplete retinal degeneration, characterized by a loss of the junction between inner and outer segments of the photoreceptor layer (IS/OS line), a relatively preserved ONL on SD-OCT, and partially remnant response on ERG (Fig. 3C–E). Three of the 10 rabbits had complete retinal degeneration including diffuse loss of the IS/OS line and ONL on SD-OCT, which implies diffuse outer retinal degeneration, and complete loss of responses on ERG (Fig. 3H–J).

**Figure 4:**
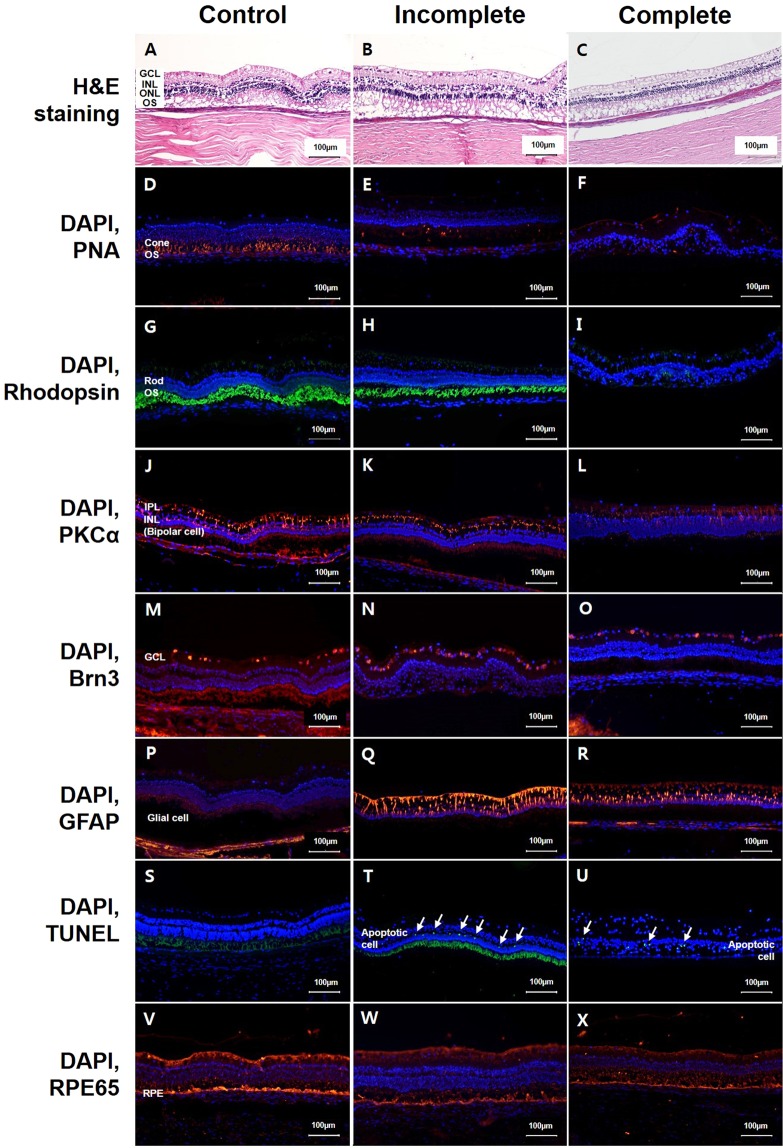
Differences in histology with H&E staining, immunohistochemistry with RPE65, PNA, rhodopsin, PKCα, Brn3, GFAP, and the TUNEL assay between control eyes (left eye of same animal) and eyes with incomplete or complete changes in retinal degeneration at six weeks after intravitreal injection of 0.4 mg of SI with vitrectomy. Histology and immunohistochemistry findings demonstrated the difference between control eyes (**A,D,G,J,M,P,S,V**), eyes with incomplete retinal degeneration (**B,E,H,K,N,Q,T,W**), and eyes with complete retinal degeneration (**C,F,I,L,O,R,U,X**). In control eyes, all layers of the retina were easily distinguished by histology (**A**), and the cone and rod photoreceptors, bipolar cells, and ganglion cells were distinctly stained by PNA, rhodopsin, PKCα, and Brn3, respectively (**D,G,J,M**). In histology with H&E staining, eyes with incomplete and complete retinal degeneration showed loss of the photoreceptor layer and retinal thinning (**B,C**). In RPE65 staining, control eyes and eyes with retinal degeneration showed intact RPE cells (**V,W,X**). In the eyes with incomplete retinal degeneration, there were fewer cone photoreceptor cells based on PNA staining (**E**) compared to control, and the rod photoreceptor cells were normal based on rhodopsin staining (**H**). However, in the eyes with complete retinal degeneration, the cone photoreceptor cells and rod photoreceptor cells were nearly depleted based on PNA staining (**F**) and rhodopsin staining (**I**), respectively. Bipolar cells remained intact based on PKCα staining (**K**), and ganglion cells were normal based on Brn3 staining (**N**) in incompletely affected eyes. However, bipolar cells and ganglion cells were slightly decreased in eyes with complete retinal degeneration (**L,O**). All eyes with retinal degeneration (incomplete or complete) demonstrated increased GFAP staining, suggesting proliferation of glial cells (**Q,R**), which was not observed in the control eyes (**P**). There were some TUNEL-positive cells in the eyes with incomplete or complete retinal degeneration (**T,U**). However, there were no TUNEL-positive cells in the control eyes (**S**).

Histology and immunohistochemistry findings demonstrated differences between the control eyes (left eye within the same animal) and SI-injected eyes with incomplete and complete changes in retinal degeneration (Fig. 4).

In the control eyes, all layers of the retina were easily distinguished by histology (Fig. 4A). Additionally, RPE, cone and rod photoreceptors, bipolar cells, and ganglion cells were all easily identified with RPE65, PNA, rhodopsin, PKCα, and Brn3 staining, respectively (Fig. 4D,G,J,M,V). In eyes with incomplete changes in retinal degeneration, H&E staining revealed a minor reduction in the photoreceptor layer, and there were fewer cone photoreceptor cells based on PNA staining (Fig. 4B,E). However, the levels of RPE cells, rod photoreceptor cells, bipolar cells, and ganglion cells remained normal (Fig. 4H,K,N,W). In eyes with complete changes in retinal degeneration, H&E staining demonstrated severe disruption of the photoreceptor layer, retinal thinning, and almost complete depletion of the cone and rod photoreceptor cells based on PNA and rhodopsin staining (Fig. 4C,F,I). Additionally, bipolar cell staining and ganglion cell staining were also decreased (Fig. 4L,O). However, the level of RPE cells remained normal (Fig. 4X). Eyes determined to have any retinal degeneration (either incomplete or complete) demonstrated increased GFAP staining, suggesting a proliferation of glial cells, which was not observed in the control eyes (Fig. 4P–R). Some apoptotic cells, as determined by the TUNEL assay, were present in eyes with incomplete or complete retinal degeneration (Fig. 4T,U). However, there were no TUNEL-positive cells in the control eyes (Fig. 4S).

Cataracts caused by contact with the instrumental lens during vitrectomy were observed in four out of 10 eyes (40.0%) during examination at six weeks after injection. We observed no signs of systemic toxicity such as weight loss or death in any rabbit.

### Quantitative comparison of 0.4-mg sodium iodate injection without or with vitrectomy

**Figure 5:**
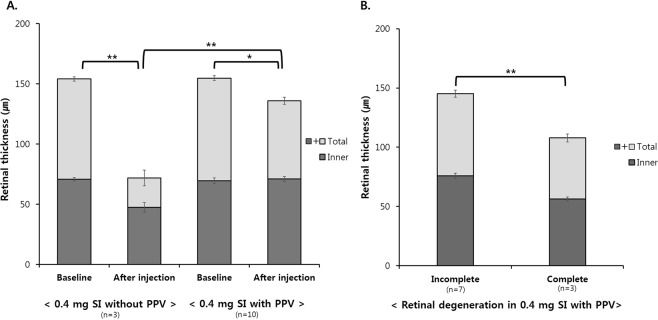
Quantitative comparison of retinal thickness after 0.4-mg SI injection without or with vitrectomy. (**A**) Following 0.4-mg SI injection in non-vitrectomized and vitrectomized rabbit eyes, total retinal thickness was significantly decreased. Compared with vitrectomized eyes, total retinal thickness was significantly thinned in non-vitrectomized eyes. In non-vitrectomized eyes, inner retinal thickness was also significantly decreased, but there was no significant change of inner retinal thickness in vitrectomized eyes. (**B**) Difference of total retinal thickness and inner retinal thickness among types of retinal degeneration with a 0.4-mg SI injection after vitrectomy. Compared with incomplete retinal degeneration, total retinal thickness and inner retinal thickness were significantly decreased in complete retinal degeneration.

We compared the effects of a 0.4-mg SI injection depending on vitrectomy. In non-vitrectomized rabbit eyes, total retinal thickness was significantly decreased after 0.4 mg SI injection (154.06 ± 1.80 μm at baseline vs. 71.80 ± 6.48 μm at one month after injection; p < 0.01; Fig. 5A). In vitrectomized rabbit eyes, total retinal thickness was also significantly decreased after injection (154.80 ± 2.08 μm at baseline vs. 135.89 ± 2.99 μm at six weeks after injection; p = 0.009; Fig. 5A). Total retinal thickness of non-vitrectomized eyes showed more thinning than did that of vitrectomized eyes (p < 0.01; Fig. 5A).

In non-vitrectomized rabbit eyes, inner retinal thickness was significantly decreased after 0.4 mg SI injection (70.83 ± 1.43 μm at baseline vs. 47.50 ± 4.00 μm at one month after injection; p < 0.01; Fig. 5A). In vitrectomized rabbit eyes, inner retinal thickness was not significantly decreased after injection (69.55 ± 2.28 μm at baseline vs. 70.95 ± 1.94 μm at six weeks after injection; p < 0.34; Fig. 5A). Inner retinal thickness of non-vitrectomized eyes presented more significant thinning than that of vitrectomized eyes (p < 0.01; Fig. 5A).

We compared total retinal thickness among the types of retinal degeneration at six weeks after 0.4-mg SI injection in vitrectomized rabbit eyes (Fig. 5B). According to severity of outer retinal degeneration, retinal thickness change was different among the groups of retinal degeneration severity. The group of complete retinal degeneration showed more significant retinal thinning than did the groups of incomplete retinal degeneration (145.25 ± 2.96 μm in total retinal layer for incomplete vs. 107.8 ± 3.35 μm in total retinal layer for complete, 75.83 ± 2.19 μm in inner retinal layer for incomplete vs. 56.3 ± 1.79 μm in inner retinal layer for complete; p < 0.01, respectively; Fig. 5B).

## Discussion

In this study, we determined that SI injection after vitrectomy was effective in inducing unilateral diffuse homogeneous outer retinal degeneration. A dose of 0.4 mg of SI in 0.05 mL of total volume was determined to be most effective and resulted in complete retinal degeneration, with loss of cone and rod photoreceptors following intravitreal injection in 30% of subjects.

We previously reported the effectiveness of vitrectomy when retinal degeneration was induced by intravitreal injection with a drug, such as N-methyl-N-nitrosourea (MNU)^19^. In the previous study, it was hard to induce diffuse outer retinal degeneration by intravitreal injection of MNU without vitrectomy. Based on our previous study, we also tried to compare the results of intravitreal SI injection without or with vitrectomy. Without vitrectomy, only localized retinal degeneration occurred in SI-injected eyes, whereas diffuse retinal degeneration was induced in SI-injected eyes after vitrectomy. When different doses of SI were tested after vitrectomy, we found that 0.5 mg of SI induced severe retinal atrophy. However, in eyes injected with 0.3 mg of SI after vitrectomy, selective outer retinal degeneration developed in only one of three eyes. Therefore, we recognized that the range for a safe and effective dose of intravitreal SI was quite narrow. Additionally, we observed that 0.4 mg of SI induced two types of retinal degeneration, and all rabbit eyes showed retinal degeneration. These results support use of the animal rabbit model induced by 0.4 mg SI injection after vitrectomy for further experiment regarding the severity of retinal degeneration. In this study, we used ultra-wide-field fundus photography and autofluorescence imaging to evaluate whether retinal degeneration was global and diffuse. Additionally, 55-degree SD-OCT images were produced to visualize retinal changes after the injections because of their wide fields of view. Importantly, the degree of outer retinal degeneration, as graded based on ERG response, was associated with SD-OCT findings and additionally validated by histological examination with immunohistochemistry.

When ERG response was partially decreased, greater damage to the IS/OS with an intact ONL was observed on SD-OCT. Finally, when all responses were lost on ERG, ONL and the photoreceptor layer were indistinguishable in SD-OCT images. Additionally, eyes with incomplete changes in retinal degeneration had fewer cone cells based on PNA staining. Eyes with complete changes in retinal degeneration, including those with flat ERG responses, also had less staining with PNA and rhodopsin. Immunohistochemistry demonstrated fewer bipolar and ganglion cells in eyes with complete changes in retinal degeneration. SI not only affects RPE cells and photoreceptors, but can also lead to toxicity and necrosis of the inner retina when intravenously injected^8,11,20^. However, in this experiment, RPE staining remained normal despite retinal degeneration compared with the control. There was no previous report about RPE staining after intravitreal injection of SI. A previous study reported morphologic changes of RPE with immunohistochemistry staining after subretinal injection of SI in other species^21^. In this report, the RPE nuclei became bigger and more rounded after subretinal injection of SI, but RPE remained relatively stable and appeared to be more resistant than the photoreceptors. The previous and present studies suggest that systemic administration of SI could induce toxicity of RPE, but localized administration of SI such as subretinal and especially intravitreal injection induce less damage to the RPE. We did find that intravitreal injection of SI after vitrectomy induced direct damage to the photoreceptor, not the level of RPE. Previous studies reported that, after SI was administered, Müller glial cells proliferated on the third day, and the ratio of GFAP-positive cells increased markedly for 28 days^8,22^. In this study, GFAP staining was also increased in all the eyes that received an intravitreal injection of SI, and it is likely that the SI injection itself caused fibrosis and proliferation of glial cells, regardless of degeneration. The TUNEL assay was positive in eyes with outer retinal degeneration, even though the degree of staining was not extensive. This could be due to a lack of apoptotic cells. SI induced the death of photoreceptors and may have triggered apoptosis. TUNEL-positive cells were restricted to the ONL, where photoreceptor nuclei are located^6,12^.

Kondo *et al*.^23^ reported a transgenic (Tg) rabbit model of progressive retinal degeneration. That rabbit model showed decreased rod ERG response in 5% of animals at 48 weeks of age. In that study, 15% of newborn rabbits were transgene-positive, and some died. In general, it is difficult to develop a genetically modified animal model, and it takes a relatively long time for disease to fully manifest. Because of these limits, intravitreal injection of compounds to induce retinal degeneration has been tried with various drugs in several different kinds of animals^24–31^. The main advantages of localized application of SI through intravitreal injection are that it spares the second eye as a control and avoids unwanted systemic side effects. With a known effective drug dose, many adequate animal models for retinal degeneration could be developed quickly. Animal models with monocular drug–induced retinal degeneration could be useful because they do not show binocular blindness. Cho *et al*.^30^ reported that intravitreal injection of SI induced monocular retinal degeneration in New Zealand white rabbit eyes. They further reported that retinal damage was reversible at low doses (0.1 and 0.2 mg of SI) but irreversible at higher doses (0.4 and 0.8 mg of SI). In the 0.4-mg SI group in that study, the outer retina was significantly destroyed, whereas the inner retina was relatively preserved. Conversely, in the 0.8-mg SI group, the entire retinal layers were irreversibly destroyed. Those authors reported that monocular intravitreal injection with SI provided an animal model for monocular retinal degeneration. However, Cho *et al*.^30^ only tested the performance of intravitreal SI injection and did not perform vitrectomy. Our study showed that intravitreal SI injection without vitrectomy did not induce diffuse retinal degeneration and produced only localized, widely varying degrees of retinal degeneration. In addition, intravitreal SI injection without vitrectomy could induce severe retinal thinning to affect the inner retinal layer. With intravitreal injection after vitrectomy, retinal degeneration was relatively uniform, and the RPE and inner retinal layer remained relatively intact per the results of immunohistochemistry and quantitative outcomes of retinal thickness. Additionally, because only conventional FP without autofluorescence imaging was used instead of ultra-wide-field FP with AF imaging, those authors were limited in their ability to determine the distribution of effects in the peripheral retina. In the present study, we demonstrated that intravitreal injection of SI after vitrectomy was an effective method to produce a globally diffuse outer retinal degeneration animal model with no systemic complications and low cost. Furthermore, if future researchers screen the degree of outer retinal degeneration before experimentation, the two types of outer retinal degeneration models induced here by SI injection can be effectively used for other study purposes.

Notably, there were some limitations in this study. First, the intravitreally injected SI dose was different between the post-vitrectomy group and the without-vitrectomy group. Because we learned that diffuse homogenous retinal degeneration in the whole retina could not be induced with intravitreal SI injection without vitrectomy in the dose-dependence study, we did not try to inject a higher SI dose than 0.4 mg. In the dose-dependent study of SI with vitrectomy, we confirmed that intravitreal injection of SI after vitrectomy affected the whole retina homogeneously based on wide AF and SD-OCT images and focused on detecting the effective dose that could induce diffuse retinal degeneration. We initially could not predict the effect of intravitreal SI injection after vitrectomy and could not rule out the possibility that even lower doses of SI might induce retinal degeneration in the vitrectomized eye. Therefore, sham and a higher dose of SI (0.5 mg) were also injected in vitrectomized eyes. Second, the follow-up periods of the dose-dependent study and the second study were different. In the dose-dependent study, the purpose was to find the effective dose to induce diffuse retinal degeneration, so rabbits were observed for four weeks after intravitreal injection. In the second study, the purpose was to evaluate the retinal change after a 0.4-mg SI intravitreal injection, so rabbits were sacrificed at six weeks after intravitreal injection. Third, the numbers of animals included were relatively small. However, with the rabbits used, we could compare the effects of vitrectomy on the induction of diffuse retinal degeneration and determine the effective dose. Furthermore, in the second study, complete retinal degeneration was induced at 30% among 10 rabbits. So, while minimizing the number of sacrificed animals, the aim of this study was achieved.

This SI-induced method provides an animal model that includes incomplete and complete outer retinal degeneration and will be useful for evaluating therapeutic strategies for diseases involving retinal degeneration. After vitrectomy, an animal model induced by intravitreal SI injection showed diffuse and uniform outer retinal degeneration.

In conclusion, a vitrectomized rabbit model with an intravitreal a 0.4-mg/0.05-mL SI injection induced diffuse outer retinal degeneration with disruption of photoreceptors.

## Materials and Methods

### Animals

In a dose-dependent study of SI, the right eyes of male New Zealand white rabbits (n = 19), aged five months and weighing between 2.5 and 3.5 kg, received either an intravitreal injection of SI without vitrectomy or an intravitreal injection of SI or sham injection at two weeks after vitrectomy. For all injections, each dose of SI was diluted in 0.05 mL of phosphate-buffered saline (PBS). Two rabbits per concentration received the following intravitreal injections of SI without vitrectomy: 0.4 mg, 0.3 mg, 0.2 mg, and 0.1 mg. Intravitreal injection of SI after vitrectomy was performed as follows: sham (0.05 mL of PBS; n = 2 rabbits) or 0.5 mg, 0.3 mg, or 0.1 mg of SI (n = 3 rabbits per concentration). To identify morphological changes to the retina, ultra-wide-field color FP, fundus AF imaging, and SD-OCT were performed at one month after intravitreal SI injection. Histological examinations after H&E staining were also completed on select rabbit eyes at one month after injection. Additionally, to analyze the change of retinal degeneration over time, SD-OCT was also performed at one week after intravitreal SI injection in the vitrectomized eyes.

Based on the results of the dose-dependence study, 0.4 mg of SI in 0.05 mL of PBS was chosen for injection into the right eyes of rabbits (n = 10) at two weeks after vitrectomy. The rabbits in the efficacy experiments had a mean body weight of 3.32 kg and mean axial length according to A-scan ultrasonography of 15.96 mm. In addition, FP, AF imaging, SD-OCT, and histology were performed at six weeks after injection. To identify physiological changes to the retina, ERG was also performed at six weeks after injection.

All procedures adhered to the Association for Research in Vision and Ophthalmology (ARVO) Statement for the Use of Animals in Ophthalmic and Vision Research (ARVO Animal Policy). Approval for this study was obtained from the Institutional Animal Care and Use Committee of Korea University College of Medicine in Seoul, Korea.

### Vitrectomy

The rabbits were anesthetized by injection of alfaxalone (5 mg/kg, Alfaxan®; Jurox Pty Ltd., Rutherford, Australia) into the marginal auricular vein and intramuscular injection of xylazine (4 mg/kg, Rompun®; Bayer AG, Leverkusen, Germany). After anesthesia, 0.5% tropicamide and 0.5% phenylephrine (Tropherine®; Hanmi Pharm Co., Ltd., Seoul, Korea) were administered for pupil dilatation, and then the eye was irrigated with 5% povidone iodide and draped for surgery. Two-port, 23-gauge core vitrectomy (Associate; DORC, Zuidland, the Netherlands) was performed with a direct biconcave lens. The two ports were prepared by inserting a trocar cannula into the sclera at 4 mm from the limbus on the superoventral and superodosal sides. A surgical microscope was used for lighting. The vitreous was removed using a vitreous cutter while continually supplying balanced salt solution (BSS; Alcon, Fort Worth, TX, USA).

### Intravitreal injection of sodium iodate

Animals were anesthetized as described above. Immediately before the injections, SI (Sigma-Aldrich, St. Louis, MO, USA) was dissolved in PBS. The right eye of each rabbit was prepared, and the corresponding dose of SI (in a total volume of 0.05 mL) was injected intravitreally at 4 mm posterior to the limbus using a 30-gauge needle. No injections were performed in the left eyes.

### Ultra-wide-field imaging and spectral-domain optical coherence tomography

FP and AF images were captured using an ultra-wide-field scanning laser ophthalmoscope (OPTOS 200 TX; Optos PLC, Dunfermline, UK). SD-OCT was performed using the Spectralis® OCT system (Heidelberg Engineering GmbH, Heidelberg, Germany). The area of the visual streak below the optic disc was evaluated. Vertical line scans, horizontal line scans, and raster scans (33 B-scans over a 16.5-mm × 16.5-mm area in a 55-degree image) were performed in high-resolution mode (1,536 A-scans per B-scan, lateral resolution = 10 µm/pixel in a 55-degree image). Up to 100 single images were averaged in automatic real-time mode to obtain a high-quality mean image.

### Electroretinography

The ERG protocol was based on the international standard for ERG from the International Society for Clinical Electrophysiology of Vision (ISCEV)^32–34^. The rabbits were anesthetized as described above, dark-adapted for 30 minutes, and their pupils were dilated. One eye per animal was studied to avoid accidental contralateral light adaptation. Light stimulation and ERG signal recording were performed with a commercial system (RETIcom; Roland Consult, Brandenburg an der Havel, Germany) using a contact lens electrode with a built-in light resource (Kooijman/Damhof ERG lens; Medical Workshop BV, Groningen, the Netherlands). The reference and ground electrodes were platinum subdermal needle electrodes. The reference electrodes were placed in the skin near the lateral canthus of the eyes, while the ground electrode was placed on the forehead between the two eyes.

### Histological examination

Immediately after euthanasia, both eyes were enucleated, immersion-fixed in Davidson’s solution for 24 hours, dehydrated, and embedded in paraffin. Sections measuring 4 µm were cut and stained with H&E. The slides were examined for pathological retinal changes using a light microscope (BX-53; Olympus Corp., Tokyo, Japan) and photographed with a digital camera (INFINITY3-1UR; Lumenera Corp., Ottawa, ON, Canada).

### Immunohistochemistry

Tissue sections were deparaffinized, rehydrated, and microwave-heated in antigen retrieval buffer [1 mM of ethylenediaminetetraacetic acid (EDTA), 0.05% Tween 20; pH: 8.0]. Sections were then blocked with 4% horse serum in PBS, followed by primary antibody incubation at 4 °C overnight. Anti-RPE65 (Invitrogen, Carlsbad, CA, USA) staining was performed following the manufacturer’s protocol. For anti-PKCα (Invitrogen, Carlsbad, CA, USA) and rhodamine-labeled anti-peanut agglutinin (PNA) (Vector Laboratories, Burlingame, CA, USA) coimmunostaining, fluorescent detection was performed with an Alexa Fluor 488–conjugated goat anti-mouse secondary antibody (Invitrogen, Carlsbad, CA, USA). For anti-PKCα and anti-rhodopsin (Rockland Immunochemicals, Pottstown, PA, USA) coimmunostaining, fluorescence detection was performed with Alexa Fluor 488–conjugated goat anti-mouse and Alexa Fluor 594–conjugated goat anti-mouse secondary antibodies (Invitrogen, Carlsbad, CA, USA). For anti-Brn3 (Santa Cruz Biotechnology, Dallas, TX, USA) immunostaining, fluorescence detection was performed with an Alexa Fluor 594–conjugated goat anti-mouse secondary antibody. Lastly, for anti-GFAP (Novus Biological, Littleton, CO, USA) staining, sections were incubated for two hours at room temperature and then for one hour with Alexa Fluor 594–conjugated goat anti-mouse secondary antibody. A deoxynucleotidyl transferase-mediated dUTP nick-end labeling (TUNEL) assay (Merck Millipore, Burlington, MA, USA) was performed following the manufacturer’s protocol. Nuclei were counterstained with 4,6-diamidino-2-phenylindole (DAPI) (AnaSpec Inc., Fremont, CA, USA). Cells stained by TUNEL were evaluated using fluorescence microscopy (T2000-U; Nikon, Tokyo, Japan).

### Retinal thickness measurement and statistical analysis for retinal thinning induced by sodium iodate injection

In each rabbit, we measured total retinal thickness and inner retinal thickness at 10 different inferior retinal sites using linear horizontal SD-OCT imaging performed at baseline and at one month after a 0.4-mg SI/0.05-mL injection in the non-vitrectomized and vitrectomized eyes, respectively. Total retinal layer was defined as from ganglion cell layer (GCL) to RPE layer and inner retinal layer was defined as from GCL to INL.

To compare the effects of SI injection on total retinal thickness and inner retinal thickness between baseline and post-injection, statistical analysis was conducted using a Wilcoxon signed-rank test. To compare the effects of vitrectomy on total retinal thickness and inner retinal thickness, statistical analysis was conducted using the Mann–Whitney U test. To compare the type of retinal degeneration after SI injection, statistical analysis was conducted using the Mann–Whitney U test or Kruskal Wallis test. Data are presented as mean ± standard error (SE). Differences were considered statistically significant at p < 0.05.
